# Tunnel Squeezing Deformation Control and the Use of Yielding Elements in Shotcrete Linings: A Review

**DOI:** 10.3390/ma15010391

**Published:** 2022-01-05

**Authors:** Xiaomeng Zheng, Kui Wu, Zhushan Shao, Bo Yuan, Nannan Zhao

**Affiliations:** 1 School of Civil Engineering, Xi’an University of Architecture and Technology, Xi’an 710055, China; gequykigeqk@163.com (X.Z.); shaozhushan@xauat.edu.cn (Z.S.); qinsxauat@126.com (B.Y.); zhaonannan@xauat.edu.cn (N.Z.); 2 Shaanxi Key Lab. of Geotechnical and Underground Space Engineering (XAUAT), Xi’an University of Architecture and Technology, Xi’an 710055, China

**Keywords:** tunnel, squeezing deformation, yielding element, ductile lining performance

## Abstract

Shotcrete lining shows high resistance but extremely low deformability. The utilization of yielding elements in shotcrete lining, which leads to the so-called ductile lining, provides a good solution to cope with tunnel squeezing deformations. Although ductile lining exhibits great advantages regarding tunnel squeezing deformation control, little information has been comprehensively and systematically available for its mechanism and design. This is a review paper for the purpose of summarizing the development history and discussing the state of the art of ductile lining. It begins by providing a brief introduction of ductile lining and an explanation of the importance of studying this issue. A following summary of supporting mechanism and benefits of ductile lining used in tunnels excavated in squeezing ground conditions is provided. Then, it summarizes the four main types of yielding elements applied in shotcrete lining and introduces their basic structures and mechanical performances. The influences of parameters of yielding elements on the supporting effect are discussed and the design methods for ductile lining are reviewed as well. Furthermore, recommendations for further research in ductile lining are proposed. Finally, a brief summary is presented.

## 1. Introduction

Deep excavation in squeezing grounds for tunnel engineers normally implies excessive tunnel convergences [1,2,3,4,5,6,7], and these rock deformations usually take slowly, sometimes lasting for one week, several months, or more than one year, after tunnel excavation is completed [8,9,10,11,12,13,14,15]. Conventional rigid tunnel shotcrete linings, where rock deformations are strictly limited, are unable to work against great overburden pressure which is triggered by considerable rock deformations [16,17], and the bad phenomenons of shotcrete falls or cracking, or even serious tunnel collapse are often observed [18,19]. In such a condition, it is almost infeasible to contain deformation energy involved by means of heavier linings [20,21,22].

In order to avoid shotcrete lining failure in deep excavation through squeezing grounds, the use of yielding elements in shotcrete lining, leading to the so-called “ductile lining”, has gradually gained more attention. In fact, at first tunnel engineers took actions to divide the shotcrete shell into several segments, where longitudinal gaps were left in advance, in order to accept considerable rock deformations without damaging shotcrete. Unfortunately, this practice led to circumferential internal forces in lining segments not being greatly transferred over these reserved gaps, consequently followed by a significant decrease of shotcrete lining resistance [23]. Then, ductile lining replacing the open gaps with yielding elements was proposed with the attention to address the problems of both acceptance of considerable rock deformations and transfer of shotcrete lining internal forces. Yielding elements show a stronger deformability than shotcrete, providing shotcrete lining with high possible resistance and able to accommodate the controlled rock deformations through their compressible deformations [24,25,26,27,28,29]. Ductile linings were first applied in the Galgenberg tunnel, Austria, in 1994, where the yielding elements consisted of groups of axially loaded steel pipes having some manufactured local weakness, and the large squeezing deformations occurring were successfully overcome by using the yielding elements in shotcrete linings [30]. Due to the great advantages of ductile lining in tunnel squeezing deformation control, many research efforts have been made to continuously develop and improve high-performance yielding elements over the past two decades [31,32,33,34,35], such as the glass fiber reinforced plastic element (FFU) [16], telescope yielding element [36], and lining stress controller element [37]. Of course, good applications of these yielding elements in shotcrete linings have been also achieved in many tunnel cases, for instance in the Tauern tunnel in Austria [38], the Lyon-Torino Base tunnel connecting France and Italy [31,39], and the Yangshan tunnel in China [32,33,40]. In Table 1, a brief summary of famous tunnels throughout the world is provided, where large squeezing deformations were satisfactorily controlled by applying ductile linings [18,25,30,31,32,33,37,38,39,40,41,42,43]. In many international conferences, including World Tunnel Congress [18,27,44,45,46], International Rock Mechanics Congress [16,36,47,48,49,50,51], and many other conferences [42,52,53,54,55], much attention has been paid and hot discussions raised on the topic of design and use of ductile linings in squeezing rock tunnels. In addition, many researchers have also attempted to investigate the influences of the limited set of design parameters on ductile lining performances or the interaction between rocks and linings, and presented their results in research article form [19,23,25,56,57,58,59,60,61,62,63,64,65]. The use of yielding elements in shotcrete linings for tunnel squeezing deformation control at first glance appears as simple work, however in practice it is rather challenging because of the time-dependent hardening of shotcrete, non-linear mechanical response of yielding elements, and the time and tunnel face-advancement dependent development of rock deformations. A more serious failure would take place remarkably if they are employed incorrectly [66,67]. However, up to now, there still has not been a systematic and comprehensive summary on previous research, which is fundamental for future research leading to a deeper understanding and better application of ductile linings.

Previous to this work, little information was comprehensively and systematically available for the mechanism and design of ductile linings in squeezing rock tunnels. This is a review paper concentrating on the development history and state of the art of ductile linings. This review article is arranged in six Sections. After the “Introduction”, the supporting mechanism and benefits of ductile linings applied in squeezing rock tunnels are explained in Section 2. Section 3 summarizes four main types of yielding elements (Highly deformable concrete element, Lining stress controller element, Wabe element, and Support resistance limiting damper) and introduces their basic structures and mechanical performances. In Section 4, the influences of parameters of yielding elements on the supporting effect are discussed and the design methods for ductile lining are reviewed. Based on authors’ experience in this research field, recommendations for further research in ductile lining are proposed in Section 5. Finally, a brief summary is proposed.

## 2. Supporting Mechanism and Benefits of Ductile Linings

**Figure 1:**
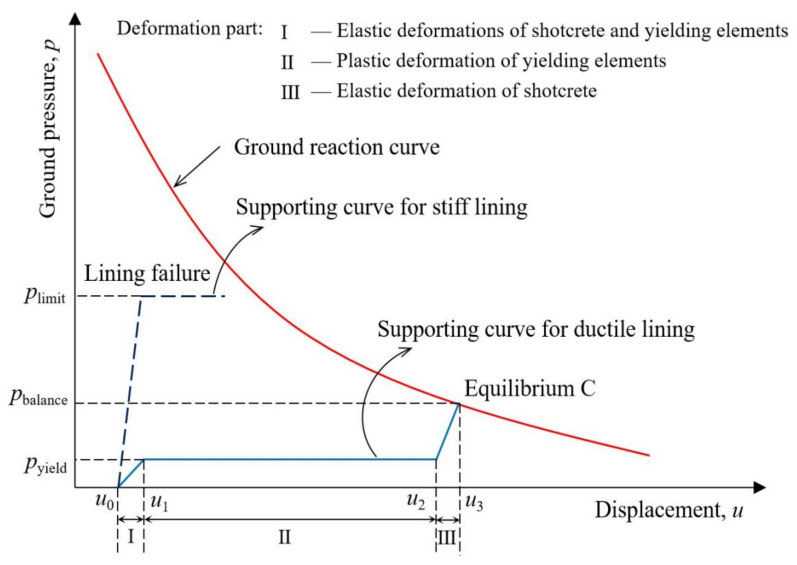
Comparison of supporting characteristic curves between stiff lining and ductile lining [35]. Reproduced with permission from [37].

The International Society for Rock Mechanisms (ISRM) has provided a qualitative definition of “squeezing rock” in that the squeezing of rock is the deformation observed over time, and is basically related to the rock creep triggered by exceeding its limit shear stress [68]. Chu et al. [69] reported that time-related deformations were possible to account for more than 70% of the total convergences of tunnels in heavy squeezing rocks. Rabcewicz [70] stated in his book that “*…for the primary supports, it is also a futile attempt to deal with high* *overburden pressure by employing stiff supports, as those would inevitably be destroyed…*”, because measures of strengthening support structures could only be effective within a certain limit [71,72,73]. As shown in Figure 1, when a large amount of rock deformation energy is accumulated, rigid shotcrete lining, however, allowing a limited rock displacement, fails to provide a sufficient support resistance, causing its final failure [74]. Instead, the idea of “flexible principle” considers that the ground pressure will decrease as rock deformation increases, and the controlled rock displacement is necessarily permitted in large squeezing deformation tunnels, thus protecting shotcrete linings from excessive pressure and failure [75]. Ductile lining behind the “flexible principle” uses the shortenings of yielding elements to accommodate rock deformations and fulfil the intention to decrease ground pressure. The schematic diagram of ductile lining is illustrated in Figure 1. The material cost is a very important factor that must be taken into consideration in the construction. Table 2 and Table 3 provide comparisons of material costs between stiff supports and ductile linings in two tunnel cases [76]. Obviously, the application of ductile lining lost up to 30% of material cost, compared with stiff supports. In other words, besides the effective control of tunnel squeezing deformations, the goal of cost saving is also achievable by using ductile linings.

The general supporting characteristic curve for ductile lining is shown in Figure 1. Clearly, it can be broadly divided into three stages [63]. The first stage should be regarded as the common elastic deformations of both shotcrete and yielding elements after ductile linings are installed because the internal forces accumulated in the linings does not exceed the yielding stress of yielding elements during this stage. However, this process does not last a long time and the element yielding stress will be easily achieved [77]. When the yielding elements yield in the second stage, the internal forces in the lining will not increase and lining pressure remains practically unchanged. In this stage, the circumferential shortenings of the lining are totally caused by the plastic deformations of yielding elements and the controlled rock displacement is accepted with a constant support resistance *p_yield_* [78]. This stage is called the yielding stage, which cleverly makes the supporting law of ductile linings basically fit with the deformation characteristics of squeezing rocks. After the gaps close, in other words, the ultimate compressive strain of yielding elements is achieved, the deformation of ductile linings proceeds into the third stage. In the third stage, the deformation behavior of ductile linings does not differ from that of conventional rigid linings, using strong resistance only provided by shotcrete to avoid further rock displacements. Finally, an equilibrium in point C, as shown in Figure 1, is obtained between the ground and ductile lining, where the rock displacement is permitted to a considerable level and ground pressure is controlled within the bearing capacity of shotcrete linings.

## 3. Main Types of Yielding Elements

**Figure 2:**
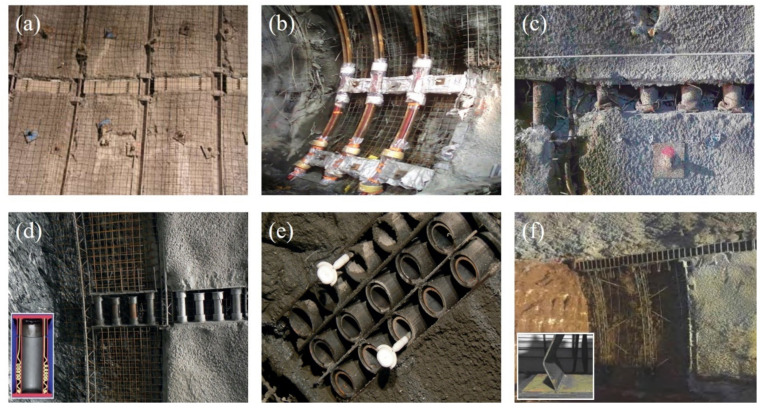
Illustration for applications of yielding elements in tunnels; (**a**,**b**) porous concrete-based element; (**c**–**e**) steel pipe-based element; and (**f**) steel plate-based element. Reproduced with permission from [37].

Over the past two decades, a series of yielding elements have been developed and improved, for instance, the FFU element [16], Meypo, DeCo-grout, Complex [25], and Telescope yielding element [36], in order to make their mechanical performances more suitable for the deformation behaviors of shotcrete and squeezing grounds. Broadly, according to their manufacturing materials, all yielding elements available can be divided into two groups: Porous concrete-based element and steel-based element [37]. A further sub-classification of steel-based element is also possible, which includes steel pipe-based element and steel plate-based element. The applications of both two types of yielding elements are shown in Figure 2, where the use of porous concrete-based elements can be seen in Figure 2a,b, steel pipe-based elements can be seen in Figure 2c–e, and steel plate-based elements can be seen in Figure 2f. In this section, the structures and mechanical properties of four yielding elements mostly used in squeezing rock tunnels are discussed in detail, including one porous concrete-based element, two steel pipe-based elements, and one steel plate-based element.

### 3.1. Highly Deformable Concrete (Hidcon) Element

Hidcon element, as shown in Figure 2a,b, is usually made of high-strength concrete matrix with porous additives [24]. Sometimes, tunnel engineers prefer to call it the “porous concrete element”. If the Hidcon element is adopted as the yielding element used in shotcrete linings, some other additives are also often used, in order to increase the compressive strength and deformability of this element [78]. Taking the Hidcon element used in the Saint Martin La Porte access adit of the Lyon–Turin Base tunnel as an example, steel fibres and hollow glass particles were applied in the elements [79]. The addition of steel fibres led to a significant improvement of element strength, and the hollow glass particles contributed to an increase of element controllable compression value, as a result of particles collapsing at a predefined compressive stress.

**Figure 3:**
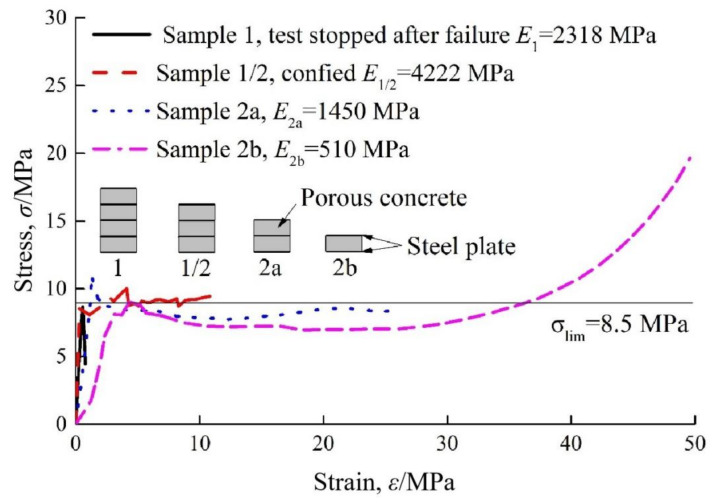
Stress-strain curves for Hidcon elements applied in the Saint Martin La Porte access adit [79]. Reproduced with permission from [79].

Typical stress-strain curves for the Hidcon element employed in the Saint Martin La Porte access adit are plotted in Figure 3. It is obvious that Hidcon elements present a high initial stiffness within a small strain range, followed by an almost unchanged resistance over a great strain range after reaching their yielding stress. The maximum strain of the Hidcon element in Figure 3 can even amount to 50%, and its resistance exhibited a high increase in the later deformation stage. Another advantage of the Hidcon element should be highlighted in that there usually does not exist a sudden brittle failure during its shortenings. However, tunnel engineers often worry about the damage of progressive hardening shotcrete when using Hidcon elements in shotcrete linings because of their high stiffness in the early deformation stage.

### 3.2. Lining Stress Controller Element

As previously mentioned, groups of axially-loaded steel pipes were first applied as the yielding elements used in shotcrete linings in the Galgenberg tunnel, Austria, in 1994 [30]. Considering the low strength of young shotcrete, those pipes featured a row of holes in order to decrease their initial stiffness. However, this type of yielding element showed a quite unstable load-displacement behavior due to the buckling of steel pipes. To overcome such a problem, tunnel engineers working at the Institute for Rock Mechanics and Tunnelling, Graz University of Technology, Austria, attempted to add shorter guiding pipes in length and insert them in those steel pipes, thus optimizing the buckling route of pipe elements [37], which is the so-called “Lining stress controller” (LSC).

**Figure 4:**
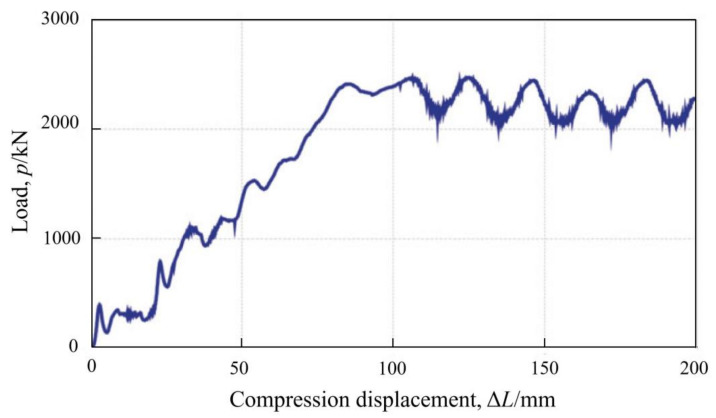
Load-displacement curve for a LSC element [37]. Reproduced with permission from [37].

Up to now, a good Lining stress controller consists of axially loaded steel pipes, where additional pipes are simultaneously installed at both ends of the element, aligned concentrically with the load-bearing pipes [24], as shown in Figure 2d. The development of load-bearing pipe buckling folds either inwards or outwards is strictly restrained due to the presence of these additional guiding pipes installed concentrically. Lining stress controller can take advantage of rationally symmetrical cylinder buckling in this way, making its load-displacement behavior better match the strength development of shotcrete. Obviously, it is very convenient to adjust the bearing capacity and allowable shortening value of the LSC element by flexibly determining the number and length of steel pipes used in the element. Figure 4 exhibits the load-displacement curve for a LSC element, where four yielding steel pipes are contained and two of them are 30 mm shorter in length. It can be seen that a practically linearly increasing load resistance is provided by the LSC element until its shortening value of 80 mm. Subsequently, its load resistance oscillates within a stable range of 2050 kN and 2500 kN, triggered by pipe buckling.

### 3.3. Wabe Element

**Figure 5:**
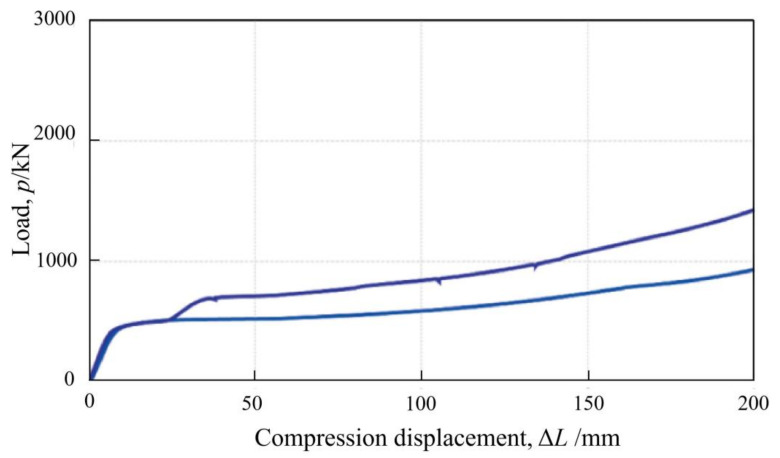
Load-displacement curve for a Wabe element [37]. Reproduced with permission from [37].

The Wabe element, compared with the LSC element, is composed of a set of transversely loaded steel pipes, which are connected with steel plates and finally bonded by top and bottom plates, as illustrated in Figure 2e. The Wabe element was first proposed and applied in the second tube of the Tauern tunnel [38]. The load-displacement curve for a Wabe element consisting of three rows of five steel pipes each, is plotted in Figure 5. It clearly shows that there is a remarkable increase of initial load resistance of the Wabe element during a very small shortening of about 8 mm, but this value in the LSC element approximately equals to 80 mm. A load resistance of about 500 kN can be provided after a compression of 10 mm is completed, which remains almost constant in the next few tens of centimeters of shortening. The element resistance presents a small increase from about the compression deformation of 80 mm, and its final value approximately equals to 900 kN with 200 mm of shortening.

Of course, it is also feasible, like the LSC element, to insert additional steel pipes that have smaller diameters to increase the load resistance of the Wabe element. It can be easily found in Figure 5 that the resistance of the Wabe element (with additional steel pipes) has an increase of about 200 kN at a shortening of 30 mm, and the final load approximates 1400 kN in this way. By comparing results in Figure 4 and Figure 5, it indicates that the resistance capacity of steel pipe-based elements is highly affected by the forced direction of steel pipes.

### 3.4. Support Resistance Limiting Damper

Support resistance limiting damper (SRLD, as shown in Figure 2f), is a type of steel plate-based yielding elements. This element is composed of upper and lower connecting steel plates and several vertical resistance-limiting plates [32,33,40]. The upper and lower connecting steel plates are placed in parallel, and the vertical resistance-limiting plates are welded on them. The vertical steel plates are produced by low-carbon steel, which has good yielding deformability and post-peak residual load-bearing capacity. By using the bending plastic deformations of these vertical steel plates, the resistance limiting element is able to achieve the purpose of releasing the deformation energy of rocks, and thus decreasing the internal forces in shotcrete linings. As reported, a huge advantage of resistance limiting elements is that they can work together with ordinary steel arches and shotcrete [32]. However, steel sets with sliding connections are usually needed for coordinate deformations, when using other yielding elements that are separated from arches.

**Figure 6:**
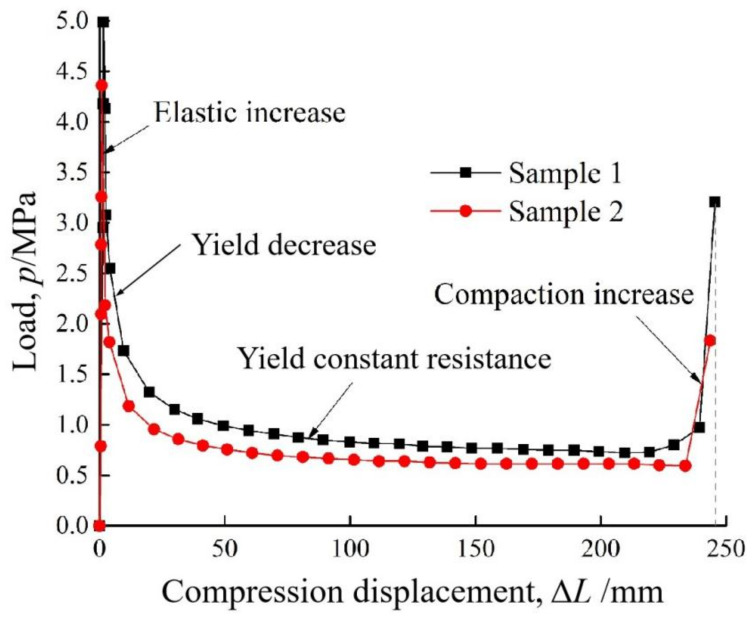
Displacement-load curve for the resistance limiting element [32]. Reproduced with permission from [37].

Figure 6 provides the load-displacement curves for two sample resistance limiting elements used in the Yangshan tunnel, in China. Obviously, the working phase of SRLD can be grouped into four stages. The first one is the elastic deformation stage, and the load resistance of SRLD increases rapidly and linearly, and reaches the peak value at an extremely small shortening in this stage. The second is the yield decrease stage, in which stage the load resistance decreases with the compression. The following is the yield constant resistance stage. The vertical steel plates generate the plastic bending in this stage, with the resistance remaining practically constant. The shortening in this stage accounts for more than 80% of the total compression value of SRLD. The last stage is the compaction rise stage and in this stage the resistance increases rapidly with the shortening. It is clearly seen from Figure 6 that SRLD has a very high initial stiffness and young shotcrete is prone to damage in the early stage. It is necessary for tunnel engineers to take action to improve this behavior performance of SRLD.

These four yielding elements above are the most commonly used types in practical engineering. In order to clearly show the difference in their performance, Table 4 lists a qualitative comparison of these yielding elements, from the aspects of several important factors, including deformability, initial stiffness, yield stress, installation procedure, serviceability, and costs.

## 4. Mechanical Performance for Ductile Linings

### 4.1. Factors Influencing the Performance of Ductile Linings

The installation quantity and location of yielding elements in shotcrete linings, reportedly, were changed several times in the Saint Martin La Porte adit, in order to fulfil the requirements of tunnel closure and shotcrete lining bearing capacity [39]. Table 5 provides these solutions used before in the tunnel. Of course, the performance of ductile lining is absolutely associated with many other factors, besides the installation quantity and location of yielding elements. Many researchers have made many efforts on this topic, in order to achieve a better understanding of the supporting mechanism and better mechanical behavior of ductile lining [80,81,82,83,84,85]. In this section, a summary of factors and how they influence the ductile lining performance is provided in detail.

Lei and Zhao [62], based on the analytical method, put forward that shotcrete linings normally suffer from the bending deformation upon loading, but shotcrete has weak resistance to such deformation due to its very low tensile strength. The ductile lining design should be based on the principle of reducing its compressive stiffness, and yielding elements are required to be installed at the place where the bending moment is relatively small. Furthermore, they have provided the calculation expressions for determining the internal forces of shotcrete lining, which can be seen in Equation (1) [62,63]. According to Equation (1), it is easy to find that when the lateral pressure coefficient does not equal to 1, the installation locations of yielding elements in shotcrete lining should be *θ* = π/4, 3π/4, 5π/4, and 7π/4, where the values of bending moments are equal to zero. In the condition of lateral pressure coefficient *λ* = 1, the bending moment value of each location in shotcrete linings is zero. In other words, a same effect will be obtained wherever yielding elements are placed in the shotcrete lining for a tunnel subjected to a hydrostatic pressure.

Interestingly, it can be found from Table 5 that tunnel engineers actually installed yielding elements in all these locations (*θ* = π/4, 3π/4, 5π/4, and 7π/4) when their quantity in shotcrete linings is not less than four. In addition, although the quantity of yielding elements was only two at chainage 1778–1784 and 1887–1915, they were both placed in the locations of *θ* = π/4 and 3π/4 in shotcrete linings.

(1) $$\left\{\begin{array}{l} M=-\left(1-\lambda \right)pr_{0}^{2}\cos\left(2\theta \right)/4\\ Q=-\left(1-\lambda \right)pr_{0}\sin\left(2\theta \right)/2\\ N=-\left[1+\lambda +\left(1-\lambda \right)\cos\left(2\theta \right)\right]pr_{0}/2 \end{array}\right.$$

where *M*, *Q*, and *N* stand for bending moment, shear force, and axial force in shotcrete linings, respectively. *p* denotes the ground pressure and *r*_0_ is the lining radius. *λ* represents the lateral pressure coefficient.

Tunnel engineers used to increase the length or quantity of yielding elements used in shotcrete linings to accept larger rock displacement because ground pressure may still be beyond the bearing capacity of shotcrete lining and its failure is possible if the total length of yielding elements is insufficient. On the other hand, however, when too many yielding elements are applied in shotcrete linings, meaning that a large rock displacement is accepted, it will lead to a high risk of tunnel collapse [85]. Reportedly, the maximum tunnel convergence had reached up to 1100 mm when the ductile linings were used in the Bolu tunnel, in Turkey, and this was an unsuccessful case regarding the application of ductile linings [86]. Therefore, how to determine the reasonable yielding element length is the key to the successful application of ductile linings in squeezing rock tunnel. Our group [60,63,87] has analytically investigated the mechanical response of ductile lining supported tunnels, and provided the theoretical solutions for rock displacement and lining pressure. Based on the analytical results, we further analyzed the influence of yield element length on tunnel time-dependent behavior. Our findings showed that there is a linear relationship between yielding element length and rock displacement (or lining pressure) in linear viscoelastic geomaterial. Rock displacement increases as element length increases while lining pressure shows an opposite trend. Our conclusion can strongly prove the point of view that it is very effective to increase yielding element length to achieve lower ground pressure and thus make it within the bearing capacity of shotcrete lining. However, under such a circumstance, excessive rock deformations possibly leading to tunnel collapse should also be given sufficient attention. Tian et al. [57,58] performed a series of numerical studies on ductile linings and suggested that if the total length of yielding element in shotcrete linings is finally determined, it will be better to select an element in shorter length, in order to obtain a more uniform lining stress distribution. However, we [63] considered that tunnel engineers must also take the construction convenience into account to finalize the yielding element length.

**Figure 7:**
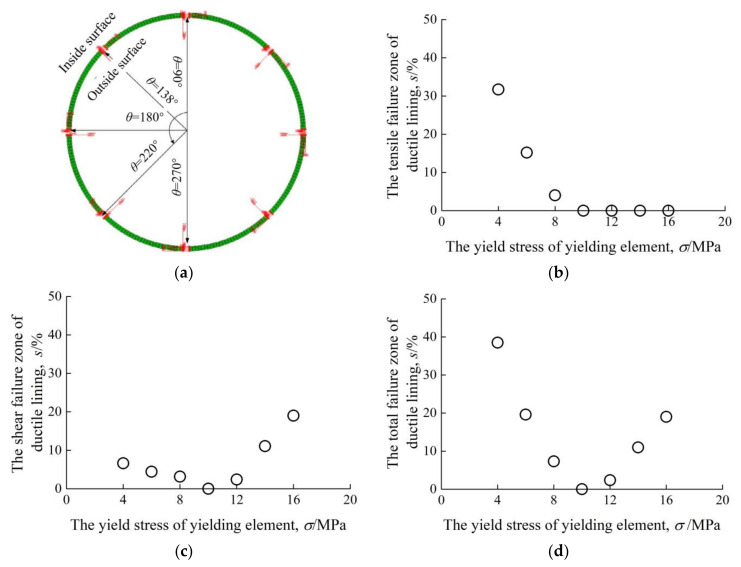
Influence of yield stress of yielding element on shotcrete lining. (**a**) Installation locations of yielding elements. (**b**) Tensile failure zone of shotcrete lining. (**c**) Shear failure zone of shotcrete lining. (**d**) Total failure zone of shotcrete lining.

Determination of yield stress of yielding elements has always been regarded as the most important and challenging technical task for tunnel engineers because of the complexity of shotcrete progressive hardening and surrounding rock relaxation. Our group found that the yield stress of yielding elements should be controlled within a reasonable range, because too large yielding stress will lead to that the elements not working before shotcrete lining damage or failure and too low yielding stress will cause an accidental rock loosening during their yield stage [60]. In addition, we [63] also concluded that when the influence of shotcrete hardening process is neglected and tunnel stability is guaranteed, there does not exist a significant difference in final rock displacement and lining pressure under different element yield stresses. Many others have reported the influence of element yield stress on the overall performance of ductile linings. Tian et al. [58], using the numerical approach, provided a statistics of failure zone in shotcrete lining in the situations of seven different element yield stresses, as shown in Figure 7. The tensile failure zone in shotcrete lining decreases with the yield stress of the yielding element, and gradually leads to zero at a yield stress of about 10 MPa, as shown in Figure 7a. Shear failure zone in shotcrete lining (see in Figure 7c) starts to decrease first as the element yield stress increases. Once the element yield stress becomes greater than 10 MPa, shear failure zone, instead, increases with yield stress. Figure 7d exhibits the development of total failure zone in shotcrete lining, including tensile and shear failure zones, and it has the same trend with shear failure zone. Generally, in this case, the optimal yield stress of the yielding element should be controlled within a range of 8 MPa and 12 MPa, which is approximately 40–60% of shotcrete compressive strength, and the minimum total failure zone in shotcrete lining can be achieved. In spite of the importance of yield stress of yielding elements on shotcrete lining performance, however, until now, related researches have not been comprehensive and thorough, and this should remain the focus for future work.

### 4.2. Design Method for Ductile Linings

The presence of yielding elements in shotcrete lining leads to, compared with conventional stiff supports, a novel support characteristic curve, as previously mentioned in Section 2. How to provide a reasonable design for ductile linings is a very important issue that tunnel engineers must face. Although several researchers have made great efforts on this topic, advancements in a ductile lining design method are not satisfactory, and there still has not been a maturely-established and universal design method for ductile lining [42,66]. Previous work on ductile lining design method can be divided into two categories, either analytical researches or numerical attempts [88,89]. In this section, a summary of breakthrough work on this issue is described in detail.

Our group have investigated the mechanical behavior of yielding elements and generally divided their deformation behavior into elastic, yield, and compaction stages. Based on the interaction between yielding elements and shotcrete linings during different deformation stages, we provided the analytical computation equations for the support characteristic curve for ductile lining [85]. As well accepted, the determination for the stiffness of lining is a crucial part for support design. However, the calculation of ductile lining stiffness is confusing now. Using the equivalent deformation principle and homogenization method, we [63] proposed a general expression for calculating the elastic modulus of ductile lining, referring to Equation (2). Furthermore, according to the deformation characteristics of yielding elements, we deduced the calculation formulas for the ductile lining stiffness in different deformation stages, as shown in Equation (3). We applied our research results to predict the time-dependent response of the Saint Martin La Porte access adit and excitingly, a successful prediction for the tunnel convergences was achieved. Radončić et al. [59], based on the convergence-confinement method, summarized the design procedure of ductile linings as six steps: 1. Determination of the equilibrium point; 2. calculation of rock displacement; 3. plotting the time-advance chart; 4. plotting the maximum support resistance curve; 5. assigning the shotcrete capacity; and 6. examination of the ductile lining stiffness. The detailed calculation process can be seen in his literature [59].

(2) $$E^{*}=\frac{E_{1}E_{2}\sum_{i=1}^{i=n}\left(l_{1i}+l_{2i}\right)}{E_{1}\sum_{i=1}^{i=n}l_{2i}+E_{2}\sum_{i=1}^{i=n}l_{1i}}$$

where E* denotes the elastic modulus of homogenized ductile lining, and *E*_1_ and *E*_2_ represent the elastic moduli of shotcrete and yielding element material, respectively. *l*_1*i*_ and *l*_2*i*_ stand for the segmental shotcrete lining and yielding element lengths, respectively.

(3) $$K_{s}^{\left(j\right)}=\left\{\begin{array}{l} \frac{E^{*}}{\left(1+\nu_{1}\right)}\cdot \frac{r_{0}^{2}-{\left(r_{0}-d_{s}\right)}^{2}}{\left(1-2\nu_{1}\right)r_{0}^{2}+{\left(r_{0}-d_{s}\right)}^{2}}\;\;(j=1)\\ 0\;\;\;\;\;\;\;\;\;\;\;\;\;\;\;\;\;\;\;\;\;\;\;\;\;\;\;\;\;\;\;\;\;\;\;\;\;\;\;\;(j=2)\\ \frac{E_{1}}{\left(1+\nu_{1}\right)}\cdot \frac{r_{0}^{2}-{\left(r_{0}-d_{s}\right)}^{2}}{\left(1-2\nu_{1}\right)r_{0}^{2}+{\left(r_{0}-d_{s}\right)}^{2}}\;\;(j=3) \end{array}\right.$$

in which *K_s_* is the homogenized ductile lining stiffness, and *ν*_1_ denotes shotcrete Poisson’s ratio. *d_s_* stands for ductile lining thickness. Based on the lining stiffness, the relationship between lining pressure (*p*) and tunnel displacement (*u*) can be written as:

(4) $$p=K_{s}\frac{u}{r_{0}}.$$

If the role of steel arches in ductile linings is considered [90], the composite ductile lining stiffness can be provided in Equation (5).

(5) $$K_{tot}=K_{s}^{\left(j\right)}+K_{sa}^{\left(j\right)}$$

where *K_tot_* represents the total stiffness of composite ductile lining, and *K_sa_* stands for steel arch stiffness. Herein, it should be noted that in case of the steel arch having function of coordinate deformation with ductile lining, its stiffness in the second stage is $K_{sa}^{\left(2\right)}=0$.

**Figure 8:**
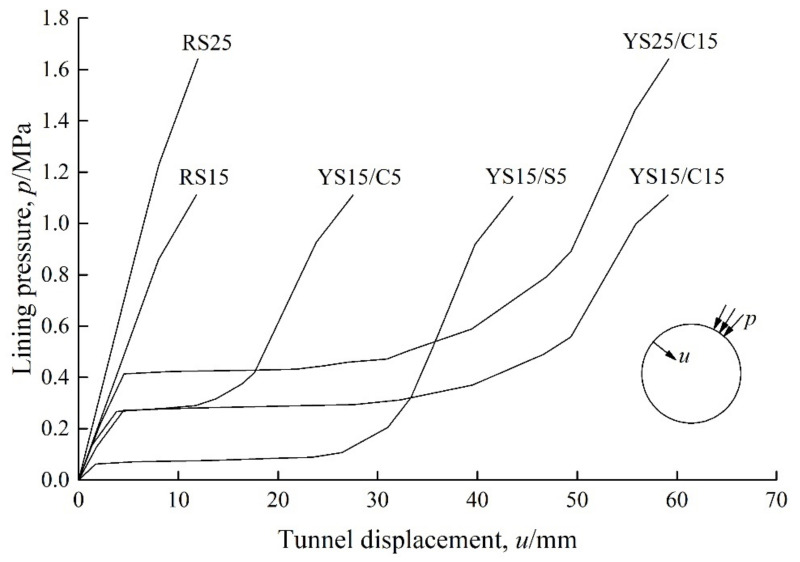
Characteristic curves for different types of tunnel linings [56]. Reproduced with permission from [56].

Ramoni and Anagnostou [56] attempted to use a numerical method to provide some supporting characteristic curves for several different types of tunnel linings, as shown in Figure 8. The numerical study is conducted in a tunnel subjected to a hydrostatic pressure, therefore, the lining pressure *p* and tunnel displacement (the radial displacement of inner contour of lining) *u* in Figure 8 keep the same in each direction. Table 6 lists the detailed components of these linings. Under the same geological conditions, results in Figure 8 well validate the supporting effect of ductile linings is significantly influenced by those factors, as mentioned previously. Based on the analytical computation equations for support characteristic curve for ductile lining, our group has successfully reproduced one of the curves in Figure 8 [85]. Of course, tunnel engineers can now easily, by using advanced computer technologies, obtain the estimation of tunnel performance in situations of different ductile lining designs. However, the premise is that there should be a preliminary design guidance, in turn providing reliable verification of the numerical design method.

## 5. Challenges and Directions for Future Research

Ductile linings have huge advantages in the tunnel squeezing deformation control. The use of yielding elements in shotcrete linings is a very challenging task because the supporting effects are influenced by many factors, and a more serious failure would happen if they are wrongly designed and applied. Up to now, although many researchers have carefully investigated ductile linings by various approaches, there still has not been a maturely-established design method for them. This greatly limits the popularization and application of ductile linings in squeezing rock tunnels. Many further researches are still needed for transferring and extending ductile lining beneficial effects into practical applications. Based on the authors’ experience in this research field, we summarize the general recommendations in following three points (1)–(3), and outline the specific research suggestions in (4)–(7).

1. Development of higher performance yielding elements, making them harmoniously work with progressive hardening shotcrete;
2. Establishment of rock quality evaluation system, making it quick and easy to judge the applicability of ductile linings in such grounds;
3. Development of universal ductile lining design method, leading to its wider applications in squeezing rock tunnels;
4. How to determine the interface interaction between rock and ductile lining, especially in the situation of anisotropic ground stress;
5. How to accurately predict the tunnel convergence with ductile lining parameters selected, so as to tunnel over-excavation in advance;
6. How to qualitatively determine the influence of ductile lining parameters on its performance, such as shotcrete hardening, yielding element installation location, and yield stress;
7. How to repair ductile linings during construction or service, with unexpected failure occurring.

## 6. Conclusions

Ductile linings show great advantages in tunnel squeezing deformation control. However, previous to this work, little information has been comprehensively and systematically available for its mechanism and design. This review paper discusses the development history and the state of the art of ductile linings. Findings in this study are summarized in the following points.

The use of yielding elements in shotcrete lining, leading to the so-called “ductile lining” is for the purpose of accepting considerable rock deformations and better use of shotcrete high resistance without damage. The deformation process of ductile lining can be generally divided into three stages. When the yielding elements yield, the circumferential shortenings of the lining are totally caused by the plastic deformations of yielding elements and the internal forces in the lining will not increase, keeping the lining pressure practically unchanged. The rock displacement is mainly released in this stage. All yielding elements can be, based on their manufacturing materials, broadly divided into two groups: Porous concrete-based element and steel-based element. Structures and mechanical performances of the four most commonly used yielding elements, HidCon, LSC, Wabe, and SRLD, are introduced, and a qualitative comparison between these four elements are provided from six aspects. The strength and initial stiffness are the most important parameters for yielding elements that an engineer should pay sufficient attention to.

Shotcrete linings usually suffer from bending deformation, but shotcrete has weak resistance due to its very low tensile strength. When the lateral pressure coefficient does not equal to 1, the optimal installation locations of yielding elements in shotcrete linings should be *θ *= π/4, 3π/4, 5π/4, and 7π/4, where bending moment values equal to zero. Rock displacement increases as yielding element length increases while lining pressure shows an opposite trend. The yield stress of yielding elements has a great influence on shotcrete failure and the yield stress of yielding elements is required to be controlled within a reasonable range, which should not be too large or too small. How to provide a reasonable design for ductile linings is still a crucial task for tunnel engineers. Previous work on ductile lining design methods can be divided into analytical studies or numerical attempts. Finally, some important recommendations for further research are outlined.

## Figures and Tables

**Table 1:** Selected tunnels employing ductile linings.

Tunnel Name	Country	Reference
Galgenberg tunnel	Austria	[30]
Semmering pilot tunnel	Austria	[41]
Strengen tunnel	Austria	[42]
Tauern tunnel	Austria	[38]
Koralm tunnel	Austria	[37]
Lyon-Torino Base tunnel	Italy	[31,39]
Ibbenbüren coal mine tunnel	Germany	[25]
Lötschberg Base tunnel	Switzerland	[18]
Ceneri Base tunnel	Switzerland	[43]
Yangshan tunnel	China	[32,33,40]

**Table 2:** Comparison of material costs in A-tunnel.

Support System	Item	Specification		Quantity/m^2^	Unit	Unit Price (Euro)	Cost (Euro)
Stiff support system	Shotcrete	1st layer	t = 250 mm, 36 N/mm^2^	17.997	m^3^	115	2070
		2nd layer	t = 200 mm, 36 N/mm^2^	13.549	m^3^	115	1558
	Steel support	1st layer	NH-200	1.996	ton	969	1934
		2nd layer	NH-150	1.170	ton	969	1134
	Rock bolt	1st layer	L = 6 m, 290 kN	21	piece	42	882
	Wire mesh	1st layer	Ground side of lining d5 mm × 150 mm spacing	51.788	m^2^	1.58	82
		2nd layer		48.596	m^2^	1.58	77
	Yielding element			-	piece	0	0
						Sum	7737
Ductile support system	Shotcrete	t = 250 mm, 36 N/mm^2^		17.341	m^3^	115	2005
	Steel support	Lattice girder		0.484	ton	920	445
	Rock bolt	L = 6 m, 290 kN		21	piece	42	882
	Wire mesh	Inner side	Both sides of lining d5 mm × 150 mm spacing	50.192	m^2^	1.58	79
		Outer side		48.197	m^2^	1.58	79
	Yielding element	LSC-N		4	piece	400	1600
						Sum	5087

**Table 3:** Comparison of material costs in B-tunnel.

Support System	Item	Specification		Quantity/m^2^	Unit	Unit Price (Euro)	Cost (Euro)
Stiff support system	Shotcrete	1st layer	t = 250 mm, 36 N/mm^2^	21.308	m^3^	115	2450
		2nd layer	t = 200 mm, 36 N/mm^2^	16.555	m^3^	115	1904
	Steel support	1st layer	NH-200	2.208	ton	1208	2450
		2nd layer	NH-150	1.361	ton	1208	1644
	Rock bolt	1st layer	L = 6 m, 290 kN	25	piece	42	1050
	Wire mesh	1st layer	Ground side of lining d5 mm × 150 mm spacing	51.309	m^2^	1.58	81
		2nd layer		47.718	m^2^	1.58	75
	Yielding element			-	piece	0	0
						Sum	9654
Ductile support system	Shotcrete	t = 250 mm, 36 N/mm^2^		20.459	m^3^	115	2353
	Steel support	Lattice girder		0.474	ton	920	436
	Rock bolt	L = 6 m, 290 kN		25	piece	42	1050
	Wire mesh	Inner side	Both sides of lining d5 mm × 150 mm spacing	49.314	m^2^	1.58	78
		Outer side		46.920	m^2^	1.58	74
	Yielding element	LSC-N		6	piece	400	2400
						Sum	6391

**Table 4:** Qualitative comparison of four mentioned yielding elements.

Criterion	HidCon	LSC	Wabe	SRLD
Deformability	Medium	High	High	High
Initial stiffness	High	Medium	Low	High
Yield stress	Medium	High	Low	Low
Installation procedure	Medium	Medium	Medium	Simple
Serviceability	Difficult	Difficult	Difficult	Difficult
Costs	Low	Low	Medium	Low

**Table 5:** Installation quantity and location of yielding elements in shotcrete linings in the Saint Martin La Porte tunnel [39]. Reproduced with permission from [39].

Chainage	1325–1444	1445–1601	1602–1747	1716–1747	174–1777
Number and position	8 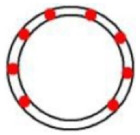	9 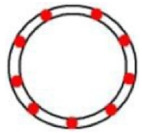	7 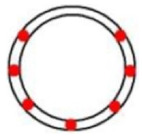	6 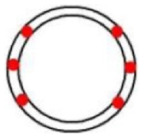	4 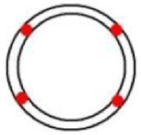
**Chainage**	**1778–1784**	**1785–1820**	**1821–1853**	**1854–1886**	**1887–1915**
Number and position	2 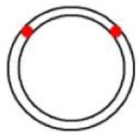	4 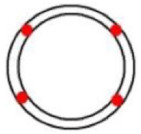	6 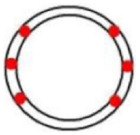	4 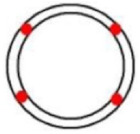	2 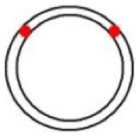

**Table 6:** Tunnel support system [56]. Reproduced with permission from [56].

Support System	Shotcrete Thickness *d*_1_/cm	Arch Type	Yielding Elements	Material	Length *d*_2_/cm	Illustration
			Number × Yielding Deformation/cm			
Rigid support	-	-	-	-	-	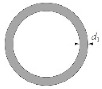
RS15	15	TH36	-	-	-	
RS25	25	TH36	-	-	-	
Ductile lining	-	-	-	-	-	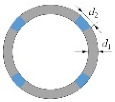
YS15/S5	15	TH36	4 × 5.0	Styrofoam	5	
YS15/C5	15	TH36	4 × 2.5	Concrete	5	
YS15/C15	15	TH36	4 × 7.5	Concrete	15	
YS25/C15	25	TH36	4 × 7.5	Concrete	15	

## Data Availability

Not applicable.

## References

[1] Tran-Manh H., Sulem J., Subrin D. (2016). Progressive degradation of rock properties and time-dependent behavior of deep tunnels. Acta Geotech..

[2] Vrakas A., Anagnostou G. (2016). Ground Response to Tunnel Re-profiling Under Heavily Squeezing Conditions. Rock Mech. Rock Eng..

[3] Wu K., Shao Z., Qin S., Li B. (2020). Determination of Deformation Mechanism and Countermeasures in Silty Clay Tunnel. J. Perform. Constr. Facil..

[4] Iasiello C., Torralbo J.C.G., Fernández C.T. (2021). Large deformations in deep tunnels excavated in weak rocks: Study on Y-Basque high-speed railway tunnels in northern Spain. Undergr. Space.

[5] Xu C., Xia C. (2021). A new large strain approach for predicting tunnel deformation in strain-softening rock mass based on the generalized Zhang-Zhu strength criterion. Int. J. Rock Mech. Min. Sci..

[6] Chu Z., Wu Z., Liu Q., Liu B., Sun J. (2021). Analytical Solution for Lined Circular Tunnels in Deep Viscoelastic Burgers Rock Considering the Longitudinal Discontinuous Excavation and Sequential Installation of Liners. J. Eng. Mech..

[7] Nistor M.M., Rahardjo H., Satyanaga A., Hao K.Z., Xiaosheng Q., Sham A.W.L. (2020). Investigation of groundwater table distribution using borehole piezometer data interpolation: Case study of Singapore. Eng. Geol..

[8] Kontogianni V., Psimoulis P., Stiros S. (2006). What is the contribution of time-dependent deformation in tunnel convergence?. Eng. Geol..

[9] Paraskevopoulou C., Diederichs M. (2018). Analysis of time-dependent deformation in tunnels using the Convergence-Confinement Method. Tunn. Undergr. Space Technol..

[10] Zhang C., Cui G., Zhang Y., Zhou H., Liu N., Huang S. (2020). Squeezing deformation control during bench excavation for the Jinping deep soft-rock tunnel. Eng. Fail. Anal..

[11] Wu K., Shao Z., Qin S., Zhao N., Chu Z. (2021). An Improved Nonlinear Creep Model for Rock Applied to Tunnel Displacement Prediction. Int. J. Appl. Mech..

[12] Arora K., Gutierrez M., Hedayat A., Cruz E.C. (2021). Time-Dependent Behavior of the Tunnels in Squeezing Ground: An Experimental Study. Rock Mech. Rock Eng..

[13] Chu Z., Wu Z., Wang Z., Weng L., Liu Q., Fan L. (2021). Micro-mechanism of brittle creep in saturated sandstone and its mechanical behavior after creep damage. Int. J. Rock Mech. Min. Sci..

[14] Hu B., Sharifzadeh M., Feng X.-T., Guo W., Talebi R. (2021). Role of stress, slenderness and foliation on large anisotropic deformations at deep underground excavations. Int. J. Min. Sci. Technol..

[15] Zhao N., Shao Z., Wu K., Chu Z., Qin S. (2021). Time-Dependent Solutions for Lined Circular Tunnels Considering Rockbolts Reinforcement and Face Advancement Effects. Int. J. Géoméch..

[16] Kurokawa S., Masumoto K., Koizumi Y., Okada Y., Utsuno M. Evaluation of deformable support in squeezing ground by experiment and numerical analysis. Proceedings of the 5th ISRM Young Scholars’ Symposium on Rock Mechanics and International Symposium on Rock Engineering for Innovative Future.

[17] Wu K., Shao Z., Sharifzadeh M., Chu Z., Qin S. (2022). Analytical Approach to Estimating the Influence of Shotcrete Hardening Property on Tunnel Response. J. Eng. Mech..

[18] Kovári K. Design methods with yielding support in squeezing and swelling rocks. Proceedings of the World Tunnel Congress.

[19] Schubert W. (1996). Dealing with squeezing conditions in Alpine tunnels. Rock Mech. Rock Eng..

[20] Ortlepp W., Stacey T. (1998). Performance of tunnel support under large deformation static and dynamic loading. Tunn. Undergr. Space Technol..

[21] Öge I.F. (2021). Revisiting the assessment of squeezing condition and energy absorption of flexible supports: A mine development case. Tunn. Undergr. Space Technol..

[22] Wu K., Shao Z. (2019). Study on the Effect of Flexible Layer on Support Structures of Tunnel Excavated in Viscoelastic Rocks. J. Eng. Mech..

[23] Lackner R., Macht J., Hellmich C., Mang H.A. (2002). Hybrid Method for Analysis of Segmented Shotcrete Tunnel Linings. J. Geotech. Geoenvironmental Eng..

[24] Radončić N., Schubert W., Moritz B. (2009). Ductile support design. Géoméch. Und Tunn..

[25] Mezger F., Ramoni M., Anagnostou G. (2018). Options for deformable segmental lining systems for tunnelling in squeezing rock. Tunn. Undergr. Space Technol..

[26] Wu K., Shao Z., Qin S., Zhao N. (2019). Mechanical analysis of tunnels supported by yieldable steel ribs in rheological rocks. Geomech. Eng..

[27] Hammer A.L., Thewes M. Integration of yielding elements in various computational methods for calculations in different planning and construction phases. Proceedings of the ITA-AITES World Tunnel Congress.

[28] Ghorbani M., Shahriar K., Sharifzadeh M., Masoudi R. (2020). A critical review on the developments of rock support systems in high stress ground conditions. Int. J. Min. Sci. Technol..

[29] Fan S., Song Z., Xu T., Wang K., Zhang Y. (2021). Tunnel deformation and stress response under the bilateral foundation pit construction: A case study. Arch. Civ. Mech. Eng..

[30] Schubert W., Brunnegger S., Staudacher R., Wenger J. (2018). Further development of yielding elements and connecting elements for shotcrete. Géoméch. Und Tunn..

[31] Barla G., Bonini M., Semeraro M. (2011). Analysis of the behaviour of a yield-control support system in squeezing rock. Tunn. Undergr. Space Technol..

[32] Qiu W., Wang G., Gong L., Shen Z., Li C., Dang J. (2018). Research and application of resistance-limiting and energy-dissipating support in large deformation tunnel. Chin. J. Rock Mech. Eng..

[33] Deng Y., Xie J., Li S. (2020). Research and Application of Support Resistant Limiting Dampers in the Deep-Buried Large-Section Loess Tunnel. Adv. Civ. Eng..

[34] Entfellner M., Hamdi P., Wang X., Wannenmacher H., Amann F. (2021). Temporary Removal: Investigating High-Strength Expanded Polystyrene (HS-EPS) as yielding support elements for tunnelling in squeezing ground conditions. Tunn. Undergr. Space Technol..

[35] Wu K., Shao Z., Qin S., Wei W., Chu Z. (2021). A critical review on the performance of yielding supports in squeezing tunnels. Tunn. Undergr. Space Technol..

[36] Verient M., Kluckner A., Radoncic N., Schubert W. Investigations on telescope yielding elements with porous filling. Proceedings of the ISRM Regional Symposium-EUROCK.

[37] Moritz B. (2011). Yielding elements—Requirements, overview and comparison/Stauchelemente—Anforderungen, Überblick und Vergleich. Géoméch. Und Tunn..

[38] Weidinger F., Lauffer H. (2009). The Tauern tunnel first and second tubes from the contractor’s viewpoint. Géoméch. Und Tunn..

[39] Bonini M., Barla G. (2012). The Saint Martin La Porte access adit (Lyon–Turin Base Tunnel) revisited. Tunn. Undergr. Space Technol..

[40] Li C., Wang G., Qiu W., Gong L., Zhao Y., Wang Q. (2020). Research and application of support resistant limiting dampers in the tunnel with high horizontal geostress. Mod. Tunn. Tech..

[41] Moritz B. (1999). Ductile Support System for Tunnels in Squeezing Rock. Ph.D. Thesis.

[42] Kolymbas D. (2005). Stress and deformation fields around a deep circular tunnel. Tunnelling and Tunnel Mechanics: A Rational Approach to Tunnelling.

[43] Merlini D., Stocker D., Falanesca M., Schuerch R. (2018). The Ceneri Base Tunnel: Construction Experience with the Southern Portion of the Flat Railway Line Crossing the Swiss Alps. Engineering.

[44] Bhavsar H., Dinis A., Fernandes E.M., Antunes P., Melâneo F. Design and construction of tunnels in zones subjected to high convergences. Proceedings of the World Tunnel Congress.

[45] Schubert W., Brunnegger S. New ductile tunnel lining system. Proceedings of the World Tunnel Congress.

[46] Hasanpour R., Hammer A.L., Thewes M. Analysis of multilateral interaction between shotcrete, yielding support and squeezing ground by means of two different numerical methods. Proceedings of the ITA-AITES World Tunnel Congress.

[47] Button E.A., Schubert W., Moritz B. The application of ductile support methods in Alpine tunnels. Proceedings of the 10th ISRM Congress.

[48] Anagnostou G., Cantieni L. Design and analysis of yielding support in squeezing ground. Proceedings of the 11th ISRM Congress.

[49] Radoncic N., Schubert W. Calculation of the shotcrete utilization for lining with integrated yielding elements. Proceedings of the ISRM International Symposium on Rock Mechanics-SINOROCK 2009.

[50] Radoncic N., Schubert W. System behaviour in weak ground: Comparison of yielding elements. Proceedings of the 12th ISRM Congress.

[51] Li C.C. Development trend of underground rock support. Proceedings of the 13th ISRM Congress.

[52] Thut A., Naterop D., Steiner P., Stolz M. Tunnelling in squeezing rock-yielding elements and face control. Proceedings of the 8th International Symposium on Tunnel Construction and Underground Structures.

[53] Schubert W. Design of ductile tunnel linings. Proceedings of the 42nd US Rock Mechanics Symposium (USRMS).

[54] Hammer A.L., Hasanpour R., Hoffmann C., Thewes M. Numerical analysis of interaction behavior of yielding supports in squeezing ground. Proceedings of the 9th European Conference on Numerical Methods in Geotechnical Engineering.

[55] Schubert W., Radoncic N. Tunnelling in “Squeezing” ground conditions-problems and solutions. Proceedings of the 13th ISRM International Congress of Rock Mechanics.

[56] Ramoni M., Anagnostou G. (2010). The Interaction Between Shield, Ground and Tunnel Support in TBM Tunnelling Through Squeezing Ground. Rock Mech. Rock Eng..

[57] Tian H., Chen W., Yang D., Wu G., Tan X. (2016). Numerical analysis on the interaction of shotcrete liner with rock for yielding supports. Tunn. Undergr. Space Technol..

[58] Tian H., Chen W., Tan X., Yang D., Wu G., Yu J. (2018). Numerical investigation of the influence of the yield stress of the yielding element on the behaviour of the shotcrete liner for yielding support. Tunn. Undergr. Space Technol..

[59] Radončić N., Schubert W. (2011). Novel method for ductile lining pre-design. Geomech. Tunn..

[60] Wu K., Shao Z., Qin S., Zhao N., Hu H. (2020). Analytical-based assessment of effect of highly deformable elements on tunnel lining within viscoelastic rocks. Int. J. Appl. Mech..

[61] Cantieni L., Anagnostou G. (2009). The interaction between yielding supports and squeezing ground. Tunn. Undergr. Space Technol..

[62] Lei S.X., Zhao W. (2020). Study on the mechanism of circumferential yielding support for soft rock tunnel with large deformation. Rock Soil Mech..

[63] Wu K., Shao Z., Qin S. (2020). An analytical design method for ductile support structures in squeezing tunnels. Arch. Civ. Mech. Eng..

[64] Gschwandtner G.G., Galler R. (2012). Input to the application of the convergence confinement method with time-dependent material behaviour of the support. Tunn. Undergr. Space Technol..

[65] Fan S., Song Z., Xu T., Zhang Y. (2021). Investigation of the microstructure damage and mechanical properties evolution of limestone subjected to high-pressure water. Constr. Build. Mater..

[66] Asef M., Reddish D., Lloyd P. (2000). Rock–support interaction analysis based on numerical modelling. Geotech. Geol. Eng..

[67] Sun Y., Bi R., Chang Q., Taherdangkoo R., Zhang J., Sun J., Huang J., Li G. (2021). Stability Analysis of Roadway Groups under Multi-Mining Disturbances. Appl. Sci..

[68] Barla G. (1995). Squeezing rocks in tunnels. Int. Soc. Rock Mech. News J..

[69] Chu Z., Wu Z., Liu Q., Liu B. (2020). Analytical Solutions for Deep-Buried Lined Tunnels Considering Longitudinal Discontinuous Excavation in Rheological Rock Mass. J. Eng. Mech..

[70] Rabcewicz L.V. (1994). Gebirgsdruck und Tunnelbau.

[71] Hoek E., Guevara R. (2009). Overcoming Squeezing in the Yacambú-Quibor Tunnel, Venezuela. Rock Mech. Rock Eng..

[72] Krastanov G., Daller J., Preh A. Tunnel design in squeezing rock conditions with high overburden. Proceedings of the 28th ITA General Assembly and World Tunnel Congress.

[73] Wu K., Shao Z., Hong S., Qin S. (2020). Analytical solutions for mechanical response of circular tunnels with double primary linings in squeezing grounds. Geomech. Eng..

[74] Barla G. (2016). Full-face excavation of large tunnels in difficult conditions. J. Rock Mech. Geotech. Eng..

[75] Wu K., Shao Z. (2019). Visco-Elastic Analysis on the Effect of Flexible Layer on Mechanical Behavior of Tunnels. Int. J. Appl. Mech..

[76] Sakai K., Schubert W. Study on ductile support system by means of convergence confinement method. Proceedings of the 5th ISRM Young Scholars’ Symposium on Rock Mechanics and International Symposium on Rock Engineering for Innovative Future.

[77] Chu Z., Wu Z., Liu B., Liu Q. (2019). Coupled analytical solutions for deep-buried circular lined tunnels considering tunnel face advancement and soft rock rheology effects. Tunn. Undergr. Space Technol..

[78] Wu K., Shao Z., Qin S. (2020). A solution for squeezing deformation control in tunnels using foamed concrete: A review. Constr. Build. Mater..

[79] Barla G., Debernardi D., Sterpi D. (2012). Time-Dependent Modeling of Tunnels in Squeezing Conditions. Int. J. Géoméch..

[80] Xu C., Xia C., Du S. (2021). Simplified solution for viscoelastic-plastic interaction between tunnel support and surrounding rock based on MC and GZZ strength criteria. Comput. Geotech..

[81] Sun Y., Li G., Zhang J., Huang J. (2021). Rockburst intensity evaluation by a novel systematic and evolved approach: Machine learning booster and application. Bull. Int. Assoc. Eng. Geol..

[82] Cebasek T.M., Likara J. (2015). A three-dimensional static numerical model of a complex underground structure in high squeezing ground. Acta Geotech. Slov..

[83] Schubert W., Moritz B. (1998). Controllable ductile support system for tunnels in squeezing rock. Felsbau.

[84] Schubert W., Radoncic N. New yielding elements for tunnel linings: Design requirements, layout and influence on system behavior. Proceedings of the ISRM International Symposium-EUROCK 2013.

[85] Wu K., Shao Z., Sharifzadeh M., Hong S., Qin S. (2021). Analytical computation of support characteristic curve for circumferential yielding lining in tunnel design. J. Rock Mech. Geotech. Eng..

[86] Dalgic S. (2002). Tunneling in squeezing rock, the Bolu tunnel, Anatolian Motorway, Turkey. Eng. Geol..

[87] Wu K., Shao Z., Qin S. (2020). Study on the interaction mechanism between surrounding rock and liner with yielding elements in squeezing tunnels. Eng. Mech..

[88] Cristescu N., Fotǎ D., Medveş E. (1987). Tunnel support analysis incorporating rock creep. Int. J. Rock Mech. Min. Sci. Géoméch. Abstr..

[89] Liu Y., Sulem J., Subrin D., Tran-Manh H., Humbert E. (2021). Time-Dependent Behavior of Saint-Martin-La-Porte Exploratory Galleries: Field Data Processing and Numerical Modeling of Excavation in Squeezing Rock Conditions. Int. J. Géoméch..

[90] Yan Q., Li S.C., Xie C., Li Y. (2018). Analytical Solution for Bolted Tunnels in Expansive Loess Using the Convergence-Confinement Method. Int. J. Géoméch..

